# Correlating pore space morphology with numerically computed soil gas diffusion for structured loam and sand, including stochastic 3D microstructure modeling

**DOI:** 10.1038/s41598-025-05825-0

**Published:** 2025-06-20

**Authors:** Benedikt Prifling, Matthias Weber, Maximilian Rötzer, Nadja Ray, Alexander Prechtel, Maxime Phalempin, Steffen Schlüter, Doris Vetterlein, Volker Schmidt

**Affiliations:** 1 Institute of Stochastics, Ulm University, Ulm, Germany; 2 Department of Mathematics, Friedrich-Alexander University of Erlangen-Nürnberg, Erlangen, Germany; 3 Mathematical Institute for Machine Learning and Data Science, Catholic University of Eichstätt-Ingolstadt, Ingolstadt, Germany; 4 Department of Soil System Science, Helmholtz-Centre for Environmental Research-UFZ, Halle, Germany

**Keywords:** Structure–property relationship, Soil gas diffusion, 3D CT data, Statistical image analysis, Stochastic 3D modeling, Computational methods, Geomorphology

## Abstract

Biogeochemical soil processes are closely linked to the structure of soil. In particular, nutrient transport depends on diffusivity and permeability within the soil’s pore network. A deeper understanding of the relationship between microscopic soil structure and such effective macroscopic properties can be obtained by tomographic imaging combined with a quantitative analysis of soil morphology and numerical simulations of effective macroscopic properties. In a previous work it has been shown that different parametric regression formulas can be used to predict these relations for finely sieved soils of loam and sand. In the present paper, we validate these formulas and further extend their applicability to structured soils. In particular, 3D CT data of a total of six samples, consisting of three loam and three sand samples, are used as the basis for an extensive structural analysis. As expected, the performance of most regression formulas can be improved by specifically adjusting their parameters for the considered soil structures. However, it turns out that some regression formulas based on, e.g., tortuosity which were fitted for finely sieved soils still reliably predict diffusion for structured soils without adjusting their parameters. For additional validation and improvement of the considered regression formulas, artificially generated soil structures can be utilized. Therefore, a method for the generation of such structures via samples drawn from a parametric stochastic 3D microstructure model is outlined which may serve as a basis for further work.

## Introduction

The three-dimensional microstructure of many natural and technical materials plays a decisive role with regard to effective macroscopic properties^1–3^. One example of an effective macroscopic property is the effective diffusivity, which is of major importance for a wide spectrum of technical applications ranging from lithium-ion batteries to solid-oxide fuel cells^4–7^. However, diffusion processes are not only of great importance in technical materials, but also in natural materials such as soils. In particular, it is well known that the 3D microstructure of the pore space has a profound impact on the transport of gas and water in soils^8,9^. While many well-established relations formulate a dependence of the scalar diffusion coefficient only on the porous medium’s porosity (see^10^ for a review of different approaches), more precise structure-property for soil gas diffusion have been recently investigated based on 3D CT data of finely sieved soils^11^. More precisely, the parameters of analytical regression formulas, which link geometrical descriptors of 3D microstructure such as tortuosity or chord length distribution and diffusive properties computed via numerical simulations, have been calibrated in^11^ to 3D image data of finely sieved loam and sand, as well as compared to structure-property relationships from the literature. The focus of the present paper is to use 3D image data of structured soils (again considering loam and sand as representatives of two different soil textures), where we first investigate to which extent the 3D morphology of structured soil differs from that of finely sieved soil. Moreover, the structure-property relationships derived in^11^ are applied to and evaluated for structured soils. In doing so, we investigate to which extent these microstructure-property relationships hold for the present data of more realistic soil structures. This allows for a deeper, quantitative understanding of soil gas diffusion, which has a significant impact on nutrient supply and plant growth^8^.

To develop even more general and robust quantitative structure-property relationships that generalize for various soil textures, stochastic 3D models can be used in order to create more comprehensive data sets for model calibration and validation than this is possible by tomographic image data. Thus, to illustrate this approach, the present paper also covers the development of a parametric stochastic 3D microstructure model, which is calibrated to 3D image data of structured sand. More precisely, excursion sets of random fields are used to model the 3D microstructure of the soil. Similar modeling ideas have been applied to generate digital twins of the 3D microstructure of lithium-ion batteries^12,13^, solid-oxide fuel cells^14–16^, and gas-diffusion electrodes^17^. Model validation is carried out by comparing geometrical descriptors of simulated 3D structures and experimental image data, which have not been used for model calibration. This lays ground for generating virtual but realistic 3D soil structures just at the cost of computer simulations by systematic variation of the model parameters.

The outline of the present paper is as follows. First, the acquisition of six structured soil samples is described in “Sample acquisition”, which are then imaged via 3D CT, see “X-ray computed tomography scanning and binarization”. Next, a brief description of the computation of diffusive properties (“Computation of diffusive properties”) and various geometrical descriptors (“Geometrical descriptors of pore space”) is given. The results of a statistical analysis of the 3D pore space morphology of structured soils, in comparison to finely sieved soils, are provided in “Statistical analysis of 3D pore space morphology”. Subsequently, quantitative structure-property relationships are investigated in “Regression formulas for the quantification of structure–property relationships”, where once again a comparison with the results obtained in^11^ is carried out. The calibration and validation of a parametric stochastic 3D microstructure model based on excursion sets of random fields is described in “Stochastic 3D microstructure modeling”. A summary of the main results achieved in the present paper and an outlook to future research activities are given in “Conclusion”.

## Materials and methods

This section is focusing on the explanation of the acquisition of six structured soil samples (“Sample acquisition”), their imaging via 3D CT (“X-ray computed tomography scanning and binarization”), the numerical calculation of diffusive properties of the underlying pore space (“Computation of diffusive properties”), and various geometrical descriptors of 3D microstructures (“Geometrical descriptors of pore space”).

### Sample acquisition

The six soil samples considered in the present paper are part of a previously published dataset on rhizosphere soil structure and its interplay with soil textures (i.e., loam versus sand)^18^. In short, the samples were taken in 2019 at the experimental station of the UFZ (Umweltforschungszentrum) in Bad Lauchstädt, Germany ($51^\circ$ $22^\prime$ $0^{\prime \prime }$ N, $11^\circ$ $49^\prime$ $60^{\prime \prime }$ E ). The structured soil has been sieved with a mesh size of ${20}\,\text {mm}$ before filling into ${75}\,\text {cm}$ deep excavation pits and subsequent compaction with a vibrating plate^19^, followed by six months of natural soil settling. Aluminum rings of ${5}\,\text {cm}$ in height and diameter were extracted at 6 and ${16}\,\text {cm}$ depths with a set-up that allows to preserve the intact soil structure of the samples. Directly after sampling, the samples were brought to the laboratory and kept at $4^{\circ }\mathrm{C}$ before X-ray CT scanning. Note that the finely sieved soil considered in^11^ has been sieved with a mesh size of ${1}\,\text {mm}$. For more information on the experimental set-up and design, the reader is referred to^18,19^.

### X-ray computed tomography scanning and binarization

X-ray CT scanning was performed with an industrial $\mu$-CT scanner (X-TEK XTH 225, Nikon Metrology) having an Elmer-Perkin 1620 detector panel (1750 $\times$ 2000 pixels). The obtained images were reconstructed into an 8-bit grayscale 3D tomogram having a voxel size of ${25}\,{\upmu \textrm{m}}$. The reconstruction was performed with a filtered back projection algorithm with the CT Pro 3D software (Nikon metrology). The conversion to 8-bit allowed saving considerable space without losing considerable information. During the 8-bit conversion, the grayscale range was normalized with a percentile stretching method. Further details regarding the number of projections, energy and current settings of the X-ray CT scanner are thoroughly given in^18^.

**Fig. 1:**
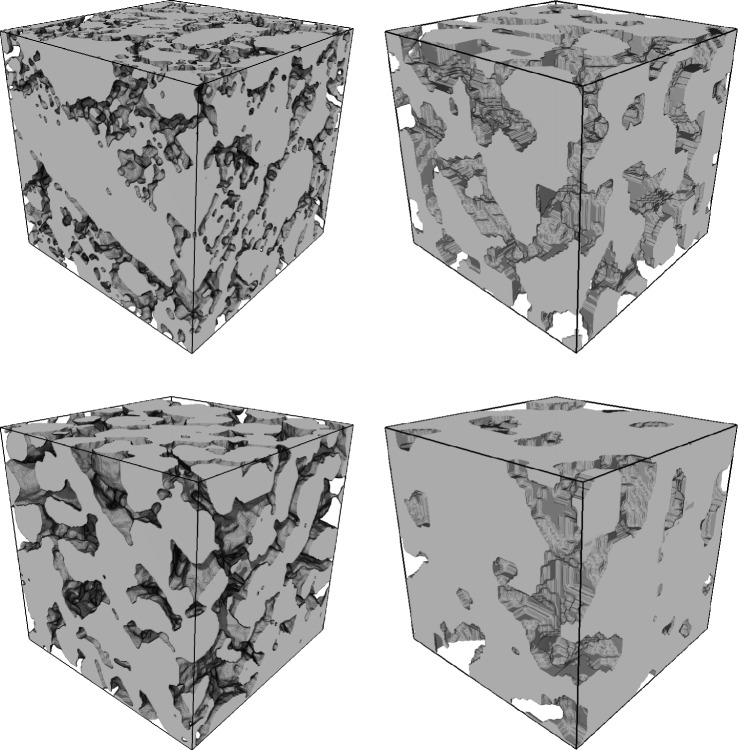
Three-dimensional renderings of selected loam (top row) and sand (bottom row) subsamples (${1.28 }\,\text {mm} \times {1.28 }\,\text {mm} \times {1.28 }\,\text {mm}$), where the solid phase is depicted in gray. Left: finely sieved soil as considered in ^11^ Right: structured soil considered in the present paper.

The segmentation into pore space, where diffusion occurs, and the soil matrix, where no diffusion can take place, was performed by a global thresholding method, see Fig. 1. For the choice of appropriate threshold values, the fuzzy c-means clustering method^20^ implemented in Quantim v.4 was used. After the phase-based segmentation, the images were cropped in cuboids with a side length of 1000 voxels in order to exclude zones of the samples which could have potentially been disturbed during sampling. The binarized 3D image data of each of the six samples was then partitioned into 446 non-overlapping cutouts such that the pore space is completely connected in *x*-direction, $y-$direction and $z-$direction, respectively. Thus, a total of 2676 cutouts was considered. The latter condition was required to ensure the convergence of the numerical simulations conducted in “Computation of diffusive properties”.

Compared to the data investigated in ^11^, the present 3D CT images differ with regard to their voxel size. While this may impede the quantitative comparison between finely sieved soil considered in ^11^ and structured soil, both datasets form a suitable basis for the development and assessment of regression formulas for the prediction of diffusion from geometrical descriptors. In particular, we will see in “Correlating pore space morphology with soil gas diffusion” that despite pronounced structural differences, caused by the different kinds of soil and the different voxel sizes, quite accurate structure-property relationships can be obtained by directly applying the regression formulas derived in^11^ to the present data of structured soil.

### Computation of diffusive properties

For each of the cutouts described in “X-ray computed tomography scanning and binarization”, effective diffusive properties are computed by the method of periodic homogenization^21^. Further details regarding these computations are described in^10,11^. As a result, we obtain a $3\times 3$ diffusion tensor $\mathbb {D} = (\mathbb {D}_{i,j})_{i,j=1,2,3}$ for each cutout. A normalized characteristic that quantifies diffusive properties is the so-called M-factor, denoted by *M*, which is defined as the ratio of effective diffusivity and intrinsic diffusivity $D_{m}$^22^. Since all soils have been sieved prior to the field experiments^18,19^ there are no pronounced anisotropy effects in the present image data. The effective diffusivity is thus calculated by averaging the diagonal entries of $\mathbb {D}$, which leads to $M = \frac{1}{3 D_{m}} (\mathbb {D}_{11} + \mathbb {D}_{22} + \mathbb {D}_{33})$.

### Geometrical descriptors of pore space

The geometrical descriptors that are used in the present paper for quantifying the 3D pore space morphology and for establishing structure-property relationships are summarized in Table 1.

**Table 1:** Overview of geometrical descriptors of pore space.

Geometrical descriptor	Symbol	Range	Unit
Volume fraction	$\varepsilon$	[0,1]	–
Specific surface area	*S*	$[0,\infty )$	$\upmu \textrm{m}^{-1}$
Mean chord length	$\mu (C)$	$[0,\infty )$	$\upmu \textrm{m}$
Characteristic bottleneck radius	$r_{\textsf{min}}$	$[0,\infty )$	$\upmu \textrm{m}$
Characteristic pore size radius	$r_{\textsf{max}}$	$[0,\infty )$	$\upmu \textrm{m}$
Constrictivity	$\beta$	[0,1]	–
Mean spherical contact distance	$\mu (H)$	$[0,\infty )$	$\upmu \textrm{m}$
Mean geodesic tortuosity	$\mu (\tau )$	$[1,\infty )$	–
Standard deviation of geodesic tortuosity	$\sigma (\tau )$	$[0,\infty )$	–

Further details on geometrical descriptors of the pore space in soils, including additional references, as well as details regarding the computation of these descriptors from voxelized 3D image data can be found in^11^. Note that the mean and standard deviation of the geometric tortuosity, which is based on the skeleton of the pore space, has been considered in^11^, but turned out to be not as useful as the concept of geodesic tortuosity with regard to the derivation of structure-property relationships. Thus, the geometric tortuosity of pore space is no longer considered in the present paper.

## Correlating pore space morphology with soil gas diffusion

In this section, we present the results of a statistical analysis of the 3D pore space morphology of structured soils, comparing them to those obtained for finely sieved soils (“Statistical analysis of 3D pore space morphology”), and we derive quantitative structure-property relationships for structured soils, where once again a comparison with corresponding relationships obtained in^11^ is carried out (“Regression formulas for the quantification of structure–property relationships”).

### Statistical analysis of 3D pore space morphology

**Fig. 2:**
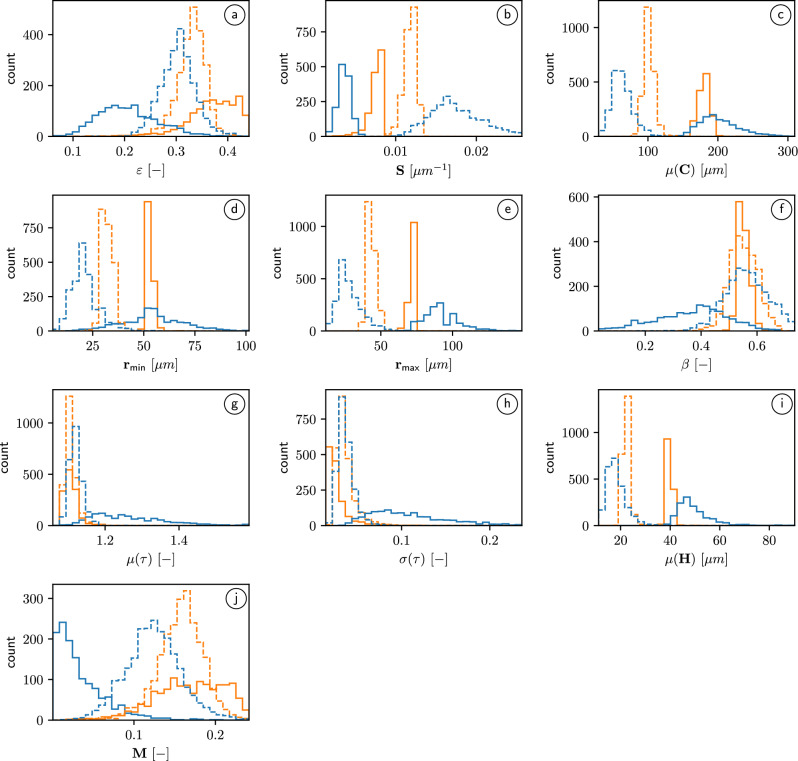
Histograms of geometrical and functional descriptors of pore space: Volume fraction (**a**), specific surface area (**b**), mean chord length (**c**), characteristic bottleneck radius (**d**), characteristic pore radius (**e**), constrictivity (**f**), mean and standard deviation of geodesic tortuosity (**g,h**), mean distance to solid phase (**i**), and the M-factor (**j**) computed from tomographic image data for all 2676 subsamples of loam (blue) and sand (orange), respectively. Solid lines represent structured soils while dashed lines correspond to finely sieved soils.

The 3D morphology of structured soil samples is investigated by means of the geometrical descriptors discussed in “Geometrical descriptors of pore space”. The obtained results are compared to those achieved in^11^ for finely sieved soils, see Fig. 2. However, note that the voxel size of tomographic image data considered in^11^ is equal to ${10}\,\upmu \textrm{m}$, whereas the image data considered in the present paper have a coarser resolution with a voxel size of ${25}\,\upmu \textrm{m}$, *i.e.*, the image data now examined for structured soils contains significantly fewer structural details than the higher-resolution image data for finely sieved soils considered in^11^. This fact has to be taken into account when comparing the results for the two sets of image data.

#### Univariate distributions of single descriptors

It turns out that the local porosities, *i.e.*, the volume fractions $\varepsilon$ of pore space computed from structured sand samples are significantly larger compared to the porosity values of structured loam, while this difference is much less pronounced for finely sieved soils, see Fig. 2a. In case of the specific surface area *S* of the pore space, significant differences between loam and sand are apparent. The values obtained for structured sand samples are significantly larger compared to those for structured loam, where the opposite is true in case of finely sieved soil, see Fig. 2b. The latter effect might be attributed to the different spatial resolutions of both types of data sets. The coarser resolution of image data considered in the present paper is also likely the reason for a significantly larger mean chord length $\mu (C)$ of the pore space (for both structured loam and sand) compared to the values obtained in^11^, since two or more chords within the pore space can now be detected as one single chord if the intermediate soil structure is too fine to be resolved with the coarser resolution. Interestingly, the pore space of the loam samples exhibits shorter mean chord lengths for finely sieved soil, whereas the opposite trend is observed with regard to structured soil, see Fig. 2c.

The coarsening effect due to a larger voxel size is also observed with regard to both characteristic radii $r_{\textsf{min}}$ and $r_{\textsf{max}}$, see Fig. 2d, e. With regard to constrictivity $\beta$, finely sieved soils showed rather similar distributions for both loam and sand, which is no longer true for structured soils. In particular, in the latter case there are pronounced differences regarding both the mean values and variances of the constrictivity distribution within each sample, see Fig. 2f. With regard to the mean geodesic tortuosity $\mu (\tau )$, both types of finely sieved soils exhibited very similar distributions with a mean around 1.1, see Fig. 2g. This is still true for structured sand, but not for structured loam, which exhibits a broad distribution of mean geodesic tortuosities. The latter distribution might arise due to the coarser resolution that is not able to resolve narrow throats within the pore phase such that the shortest paths within the pore space now consist of significantly more detours. This would also explain the plots in Fig. 2h showing the local distributions of $\sigma (\tau )$, where again the structured loam behaves differently compared to structured sand as well as both finely sieved soil types. In case of the mean distance $\mu (H)$ to the solid phase, the behavior is similar to the mean chord length $\mu (C)$, see Fig. 2i, which is intuitively clear since both geometrical descriptors quantify some kind of typical pore size.

The distributions of the local M-factor of structured soils show pronounced differences compared to those of finely sieved soils, see Fig. 2j. More precisely, both types of structured soil show broader distributions of the local M-factor. Moreover, the distribution of the M-factor corresponding to loam is now skewed to the left with a majority of values below 0.05, indicating severe limitations for soil gas diffusion within the pore space. This is likely to be caused by the significant shift of the distribution of the (local) mean geodesic tortuosity $\mu (\tau )$ to the right compared to finely sieved loam.

Overall, these investigations highlight the diversity in the descriptors depending on soil type and preparation. This observed diversity is beneficial for the development of generalized prediction formulas.

#### Bivariate distributions of descriptor pairs

**Fig. 3:**
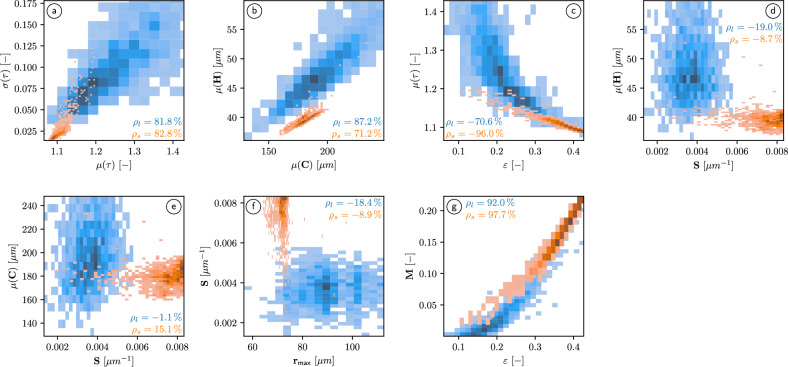
Bivariate probability densities for pairs of geometrical and functional descriptors, visualized as contour plots based on kernel density estimation, which have been obtained for the 2676 cubic subsamples of either structured loam (blue) or structured sand (orange). Furthermore, the values of Pearson’s correlation coefficient $\rho$ are given for each descriptor pair, separately computed for loam and sand.

Besides the univariate distributions discussed so far, we also consider bivariate distributions of selected pairs of geometrical and functional descriptors, see Fig. 3.

It turns out that there is nearly no correlation between the specific surface area of the pore space *S* on the one hand, and $\mu (H)$, $\mu (C)$ and $r_{\textsf{max}}$ on the other hand, see Fig. 3d–f. Note that in case of sand, this is similar to finely sieved soil, but the pronounced positive correlation between *S* and the other three geometrical descriptors mentioned above that has been observed in^11^ for finely sieved loam is no longer observed for the present data. Moreover, there is a pronounced positive correlation between $\mu (\tau )$ and $\sigma (\tau )$, which is similar for loam and sand, see Fig. 3a. Note that there is also a pronounced positive correlation between $\mu (C)$ and $\mu (H)$, where this correlation is larger for structured loam compared to structured sand, see Fig. 3b. However, in case of finely sieved soils, this correlation has been even more pronounced for both loam and sand^11^. The most pronounced correlations can be observed between the porosity $\varepsilon$ and the mean geodesic tortuosity $\mu (\tau )$ as well as between $\varepsilon$ and the M-factor *M*, see Fig. 3c, g. In both cases, the strongest correlation is observed for structured sand with correlation coefficients of $-0.96$ and 0.977, respectively. In particular, the correlation coefficient between $\varepsilon$ and *M* for structured sand, which is close to one, indicates that analytical formulas for the prediction of *M* from geometrical descriptors that only use the porosity $\varepsilon$ as input are likely to lead to decent prediction accuracy. However, as will be shown in later in “Regression formulas for the quantification of structure–property relationships”, combining the porosity with further more sophisticated geometrical descriptors allows for an even more accurate prediction of the M-factor.

### Regression formulas for the quantification of structure–property relationships

We now investigate the question how well do the prediction formulas for the M-factor derived in^11^ for finely sieved soil behave when they are directly applied to structured soil. Furthermore, we show how do the regression coefficients change compared to finely sieved soil when they are newly calibrated to the present tomographic image data measured for structured soil. An overview of the analytical prediction formulas for the M-factor considered in the following is given in Table 2.

**Table 2:** Overview of the considered analytical prediction formulas for the M-factor, including the constraints on the coefficients as well as the numerical values for the coefficients, which have been derived in^11^ for finely sieved soil.

Formula	Eq. number	Constraints	Values derived in^11^
$\widehat{M}_{1} = \varepsilon ^{c_{1}}$	(1)	$c_{1}\ge 1$	1.71
$\widehat{M}_{2} = c_{1} \varepsilon ^{c_{2}}$	(2)	$c_{1}, c_{2} > 0$	(1.92, 2.29)
$\widehat{M_{3}} = c_{1} e^{c_{2} \varepsilon }$	(3)	-	(0.02, 6.88)
$\widehat{M}_{4} = \varepsilon ^{c_{1}} \beta ^{c_{2}} \mu (\tau )^{c_{3}}$	(4)	$c_{1}>, c_{2}\ge 0, c_{3}\le 0$	(1.06, -0.04, -7.28)
$\widehat{M}_{5} = \varepsilon ^{c_{1} + c_{2}\beta } \mu (\tau )^{c_{3}}$	(5)	$c_{1}+c_{2}\ge 0, c_{3}\le 0$	(1.17, -0.18, -7)
$\widehat{M}_{6} = c_{1} \mu (\tau )^{c_{2}} \sigma (\tau )^{c_{3}} \varepsilon ^{c_{4}}$	(6)	-	(2.19, -6.07, 0.07, 1.63)
$\widehat{M}_{7} = \varepsilon ^{c_{1}} S^{c_{2}} r_{\textsf {max}}^{c_{3}}$	(7)	-	(2.6, -0.61, -0.45)
$\widehat{M}_{8} = \varepsilon ^{c_{1}}\left( \frac{\mu (C)}{\mu (H)}\right) ^{c_{2}}$	(8)	-	(2.23, 0.16)

**Fig. 4:**
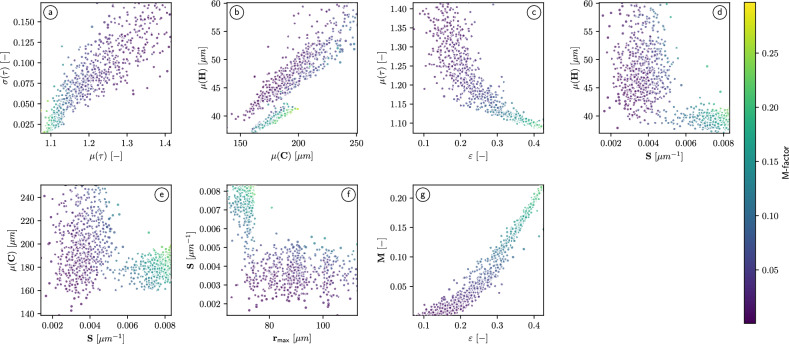
Scatter plots visualizing the dependence of the M-factor on various pairs of geometrical descriptors. The (brighter or darker) colors of the data points indicate whether the value of the corresponding M-factor is high or low. Note that in this figure we do not distinguish between the two soil textures which can be seen in Fig. 3. For clarity of presentation, only randomly selected $10\%$ of the 2676 cubic subsamples of structured loam and sand have been used to generate the 461 data points in each subfigure.

To obtain a first impression regarding the influence of geometrical descriptors of the pore space on the M-factor for structured soil, the scatter plots shown in Fig. 4 illustrate the dependence of the M-factor on various pairs of geometrical descriptors.

**Fig. 5:**
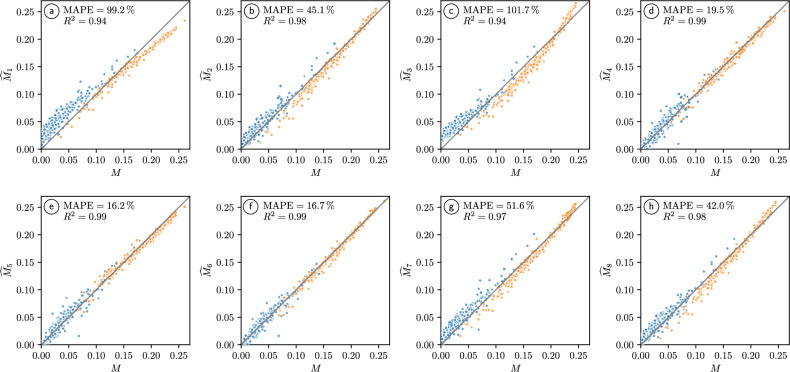
Scatter plots visualizing the values obtained for the M-factor *M* by numerical simulations versus those of the predicted M-factors $\widehat{M}_i, \, i=1,\ldots ,8$, obtained for the test data of either structured loam (blue) or structured sand (orange). The parameters appearing in the prediction formulas given in Eqs. (1)$-$(8) have been fitted to the entire training data for loam and sand, see Table 1 in the Suppl. Appendix.

Moreover, we consider several empirically derived regression formulas which will be used for the quantification of structure-property relationships and, in particular, for predicting the M-factor from the knowledge of an appropriately chosen vector of geometrical descriptors of the 3D pore space morphology. A detailed evaluation of the performance of the regression formulas and their parameters when fitted to the data of structures soils can be found in the Suppl. Appendix. For an overview, we refer to Fig. 5. In summary, the formula given in Eq. (6), which was the best-performing regression formula in ^11^, as well as the formulas given in Eqs. (4) and (5) generalize (using the parameters originally derived in ^11^) to tomographic image data of structured soils, see Fig. 5 in the Suppl. Appendix. The fit of these formulas can be further increased by adjusting their parameters specifically for structured soils, see Fig. 4 in the Suppl. Appendix. In both cases, the formula given in Eq. (6) is still the best-performing or close to the best-performing formula. In particular, the prediction accuracy of rather simple prediction formulas, which only include the porosity as geometrical descriptor (i.e. the formulas in Eqs. (1), (2) and (3)) can be increased by incorporating the mean and standard deviation of geodesic tortuosity. Furthermore, the formulas that are solely based on porosity are less robust in a sense that using that different data sets (either finely sieved soil instead of structured soil or loam instead of sand) can not be described by those equations without re-calibrating the coefficients, see Figs. 4 and 5 in the Suppl. Appendix. Interestingly, Eq. (7) is not the best-performing prediction formula despite a correlation coefficient of 0.94 between the specific surface area and the M-factor. Moreover, Eqs. (4) and (5), which both are based on the porosity, mean geodesic tortuosity and the constrictivity, are also performing well, even when using the coefficients obtained for finely sieved soil and applying them to structured soil, see Fig. 5 in the Suppl. Appendix.

## Stochastic 3D microstructure modeling

As measured CT images are time and cost-intensive, they can only provide a limited amount of data – both in terms of a rather small number of measurements as well as a comparatively small structural variety. Thus, prediction formulas which are developed solely based on measured image data may have limited explanatory power for other soil textures. In the following, we present a stochastic 3D model for the microstructure of soils which may be used to generate a large variety of realistic soil structures to overcome the limitations of measured image data. More precisely, a parametric 3D model is calibrated to tomographic image data of structured sand. By a quantitative comparison of geometrical descriptors computed on measured as well as simulated soil structures, it is shown that the complex 3D morphology of structured sand can be characterized by a surprisingly small number of interpretable model parameters. Thus, the stochastic 3D model proposed in this section lays ground for future investigations, including virtual scenario analyzes by systematic variation of model parameters.

### Model description

The modeling approach described in this section is motivated by the observation that the 3D image data of structured sand mainly shows two different classes of particles, namely a few large and many small ones. The stochastic microstructure model can be decomposed into three steps. First, we consider two independent Gaussian random fields $X_1=\{X_{1}(t), t\in \mathbb {R}^{3}\}$ and $X_2=\{ X_{2}(t), t\in \mathbb {R}^{3}\}$, where both random fields are assumed to be stationary and isotropic such that their distributions are uniquely characterized by two mean values $\mu _{1},\mu _2\in \mathbb {R}$ and two covariance functions $C_{1},C_2:[0,\infty ) \rightarrow \mathbb {R}$. Furthermore, we assume that the random fields $X_1$ and $X_2$ are normalized, i.e., it holds that $\mu _{1}=\mu _{2}=0$ and $C_{1}(0)=C_{2}(0)=1$, and that their covariance functions are given by

$$C_i(r) = \frac{2^{1-\nu _i}}{\Gamma (\nu _i)}\left( \sqrt{2\nu _i}\frac{r}{\rho _i}\right) ^{\nu _i}K_{\nu _i}\left( \sqrt{2\nu _i}\frac{r}{\rho _i}\right) ,$$

for each $r\ge 0$, where $\nu _{i} > 0$ and $\rho _{i} > 0$ are some parameters for $i \in \{1,2\}$. Here, $\Gamma$ and $K_{\nu _i}$ denote the Gamma function and the modified Bessel function of the second kind, respectively^23^. This parametric family of covariance functions is known as the Matérn covariance function, see for example Equation (4.14) in^24^, and offers a reasonable amount of flexibility via the two parameters $\nu _{i} > 0$ and $\rho _{i}$. Further frequently used covariance functions can be found in Table 15.1 in^23^. Note that the excursion set of a Gaussian random field with a slowly decreasing covariance function results in rather coarse structures, whereas the excursion set of a Gaussian random field with a quickly decreasing covariance function is able to model fine structures.

Next, for $i \in \{1,2\}$, we consider the random sets $\Xi _i = \{ t \in \mathbb {R}^{3} : X_{i}(t) \ge a_i \}$ as level sets (also called excursion sets) of the random fields $X_1$ and $X_2$, where $a_1,a_2 \in \mathbb {R}$. Due to the Gaussian nature of the random fields $X_1$ and $X_2$, the following analytical relationship between the volume fraction $\varepsilon _i=\mathbb {P}(o\in \Xi _i)$ of  $\Xi _{i}$, where $o\in \mathbb {R}^3$ denotes the origin, and the threshold $a_{i}$ is true for $i\in \{1,2\}$. Namely, it holds that $\varepsilon _{i} = 1 - \Phi (a_{i})$ for $i\in \{1,2\}$, where $\Phi :\mathbb {R}\rightarrow [0,1]$ denotes the cumulative distribution function of the univariate standard normal distribution^25^.

Finally, we model the solid phase of structured soils as union $\Xi = \Xi _1 \cup \Xi _2$ of the level sets $\Xi _1$ and $\Xi _2$. Note that the independence of $X_1$ and $X_2$ implies that the random fields $\Xi _1$ and $\Xi _2$ are also independent. Thus, the volume fraction $\varepsilon =\mathbb {P}(o\in \Xi )$ of $\Xi$ is given by $\varepsilon = \varepsilon _1 + \varepsilon _2 - \varepsilon _1 \varepsilon _2$.

### Model calibration

To fit the model described in “Model description” to measured 3D image data, note that the volume fraction $\varepsilon$ of the solid phase can be easily estimated by voxel counting^25^. Thus, $\varepsilon$ is known and can be used to exploit the relationship $\varepsilon _2 = \frac{\varepsilon - \varepsilon _1}{1 - \varepsilon _1}$, which allows us to express $\varepsilon _{2}$ in dependence of $\varepsilon _{1}$. Furthermore, this equation can be transformed to obtain the following relationship between the thresholds $a_{1}$ and $a_{2}$:

9 $$\begin{aligned} a_{2} = \Phi ^{-1}\left( \dfrac{1-\varepsilon }{\Phi (a_{1})}\right) \;. \end{aligned}$$

**Fig. 6:**
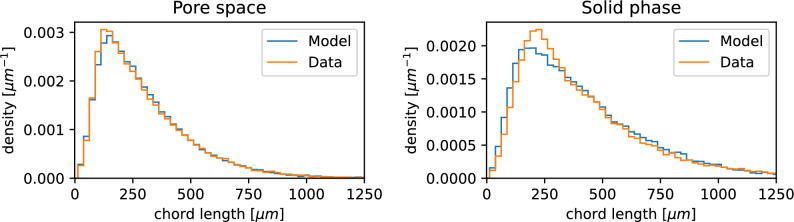
Probability density of the chord length distribution of pore space (left) and solid phase (right). In both graphs, image data of structured sand is considered, where orange corresponds to measured data and blue to a model realization.

This means that model calibration can be performed by means of the reduced parameter vector $\theta = (a_1, \nu _1, \rho _1, \nu _2, \rho _2)$, since $a_2$ is obtained from $a_{1}$ via Eq. (9), where the parameter vector $\theta$ is chosen such that simulated 3D data drawn from the calibrated stochastic model are statistically similar to the observed sand structures. More precisely, the parameter vector $\theta$ is fitted such that the chord length distributions within the solid phase as well as within the pore space match between measured and simulated data, as shown in Fig. 6.

Note that the underlying optimization procedure uses a cost function which is given by the sum of the quadratic distances between the probability densities of chord lengths of the solid phase and the pore space of measured and simulated structured sand, respectively. This cost function is chosen such that both pore and solid phase are taken into account equally. The cost function is numerically minimized via simulated annealing^26^, leading to the model parameter $\theta ^{*} = (0.04, 44.6, 70.1, 82.1, 2.86)$. For model fitting and the generation of artificial soil structures, the Gaussian random fields $X_1$ and $X_2$ introduced in “Model description” are simulated via the Fourier-based method described in^27^.

### Model validation

**Fig. 7:**
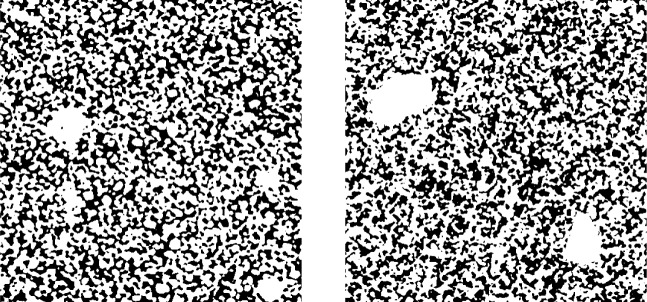
2D cross-sections of tomographic data of finely sieved sand (left) and a realization of the calibrated 3D microstructure model (right), where the white and black phase corresponds to the solid phase and the pore space, respectively.

For a structural validation of the stochastic 3D model introduced in “Model description” , we used the following approach. First, we have fitted the model in “Model calibration” to the complete tomographic image data (*i.e.*, not the small cutouts, which have been used for establishing structure-property relationships) of one of the two datasets of structured sand described in “Sample acquisition”. Then, we simulated 3D structures of the same size using the fitted model described in “Model description” . A visual comparison between tomographic image data and model realizations for structured sand is shown in Fig. 7. Finally, we computed some of the geometric descriptors stated in “Geometrical descriptors of pore space”, with the exception of mean chord lengths which were used for model calibration. As can be seen from Table 3, the simulated structures resemble the measured data quite well in terms of the considered geometric descriptors. This indicates the feasibility of further investigations of microstructure-property relationships by means of artificially generated soil structures.

**Table 3:** Overview of the geometrical descriptors computed for the pore space of measured and simulated data of structured sand.

Descriptor	Unit	Measured	Simulated
Volume fraction $\varepsilon$	–	0.586	0.583
Specific surface area *S*	$\upmu {\textrm{m}}^{-1}$	0.00215	0.00212
Mean geodesic tortuosity $\mu (\tau )$	–	1.090	1.098
Standard deviation of geodesic tortuosity $\sigma (\tau )$	–	0.0067	0.0096
Constrictivity $\beta$	–	0.514	0.452

## Conclusion

In the present paper, 3D image data of structured soils, obtained by X-ray computed tomography, is used for establishing quantitative structure-property relationships as well as for calibrating a simple stochastic 3D model for structured sand, which is based on excursion sets of Gaussian random fields.

First, tomographic image data of the 3D morphology of structured soils, which consists of three loam and three sand samples, is investigated quantitatively by means of univariate and bivariate probability distributions of geometrical and functional descriptors. These distributions are compared to those of the 3D structure of finely sieved soils, which have been considered in^11^. Similar to the situation observed for finely sieved soils, significant differences between loam and sand are apparent within the structured soils. Moreover, differences between finely sieved and structured soils are found which can be partially attributed to a different resolution of the tomographic image data. Especially for the computed M-factors, the difference between loam and sand is increased in structured soils compared to finely sieved soils.

In a second step, quantitative structure-property relationships are established via analytical regression formulas using a total of 2676 non-overlapping cutouts. For this purpose, the method of periodic homogenization is used to obtain diffusive transport properties. The regression formulas which were already established in ^11^ are used to predict the M-factor of pore space from geometric descriptors. It turns out that the previously best-performing parametric regression formula outperforms other regression formulas also when fitted on the novel image data of structured soils. Although huge differences were observed between the values of geometrical descriptors and M-factors computed from image data of structured soils compared to those obtained for finely sieved soils, this formula reliably predicts the M-factor for data of structured soils, even when used with the parameters previously derived in ^11^ for finely sieved soils. This suggests a universal applicability of this type of regression formula to various different types of structures without the need for adjustment of parameters.

To validate the applicability of the regression formulas considered in the present paper for further types of soils, stochastic 3D models may be used to generate digital twins of real soils, *i.e.*, artificial soils may be created in silico. This is an interesting direction for future research activities to obtain even more general microstructure-property relationships for various soil types. In the present paper, a first step in this direction is taken by a stochastic 3D model which is calibrated to image data of structured sand, where the solid phase is modeled as the union of excursion sets of two independent Gaussian random fields. While being a relatively simple model, validation of the model with respect to various geometric descriptors of structured sand yields promising results. This encourages continued development of stochastic 3D models for more complex soil morphologies, like those of structured loam. In particular, structured loam does not exhibit two clear length scales, which can be modeled via excursion sets of two independent Gaussian random fields.

Moreover, stochastic 3D microstructure modeling lays ground for investigating the time-dependent evolution of the 3D microstructure. More precisely, a 4D model can be obtained by separately calibrating the presented parametric 3D model to each 3D image of a time-resolved data set. This allows for predictive simulations by interpolation of the time-dependent model parameters and offers the possibility of investigating to which extent the coefficients and accuracy of microstructure-property change over time.

## Supplementary Information

Supplementary Information.

## Data Availability

The data is available upon reasonable request via an e-mail to benedikt.prifling@uni-ulm.de.
