# Supplementary material for: Correlating pore space morphology with numerically computed soil gas diffusion for structured loam and sand, including stochastic 3D microstructure modeling
Source: Sci Rep. 2025 Jun 20;15:20174. doi: 10.1038/s41598-025-05825-0 (PMC12181360; doi:10.1038/s41598-025-05825-0)
Supplement: Supplementary file 1 — Supplementary Information. [file 41598_2025_5825_MOESM1_ESM.pdf]

# Supporting Information for Correlating pore space morphology with numerically computed soil gas diffusion for structured loam and sand, including stochastic 3D microstructure modeling

Benedikt Prifling[1,*], Matthias Weber[1,*], Maximilian Rötzer[2], Nadja Ray[3], Alexander Prechtel[2], Maxime Phalempin[4], Steffen Schlüter[4], Doris Vetterlein[4], Volker Schmidt[1]

May 14, 2025

* Both authors contributed equally.
1 Institute of Stochastics, Ulm University, Ulm, Germany.
2 Department of Mathematics, Friedrich-Alexander University of Erlangen-Nürnberg, Erlangen, Germany.
3 Mathematical Institute for Machine Learning and Data Science, Catholic University of Eichstätt-Ingolstadt, Ingolstadt, Germany.
4 Department of Soil System Science, Helmholtz-Centre for Environmental Research – UFZ, Halle, Germany.

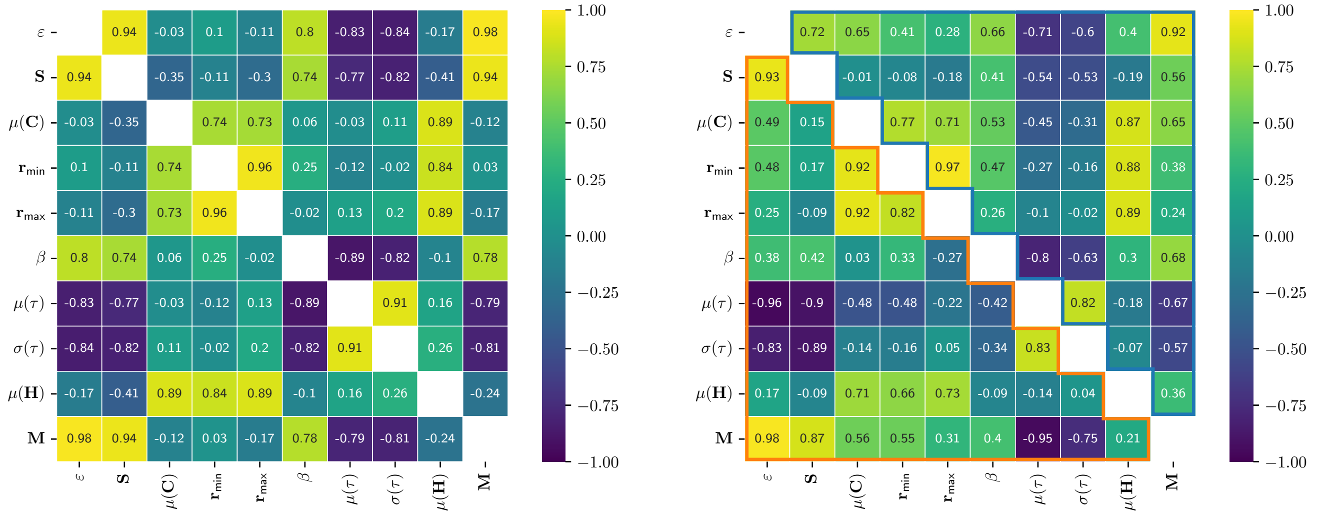


Figure 1: Correlation coefficients between all pairs of geometrical descriptors (as well as the M-factor) considered in the present paper, computed for the set of all $2 \cdot 3 \cdot 446 = 2676$ subsamples (left) and for loam and sand individually (right). In the latter case, the upper right part of the matrix (blue frame) shows the values obtained for loam, the bottom left part (orange frame) those for sand.

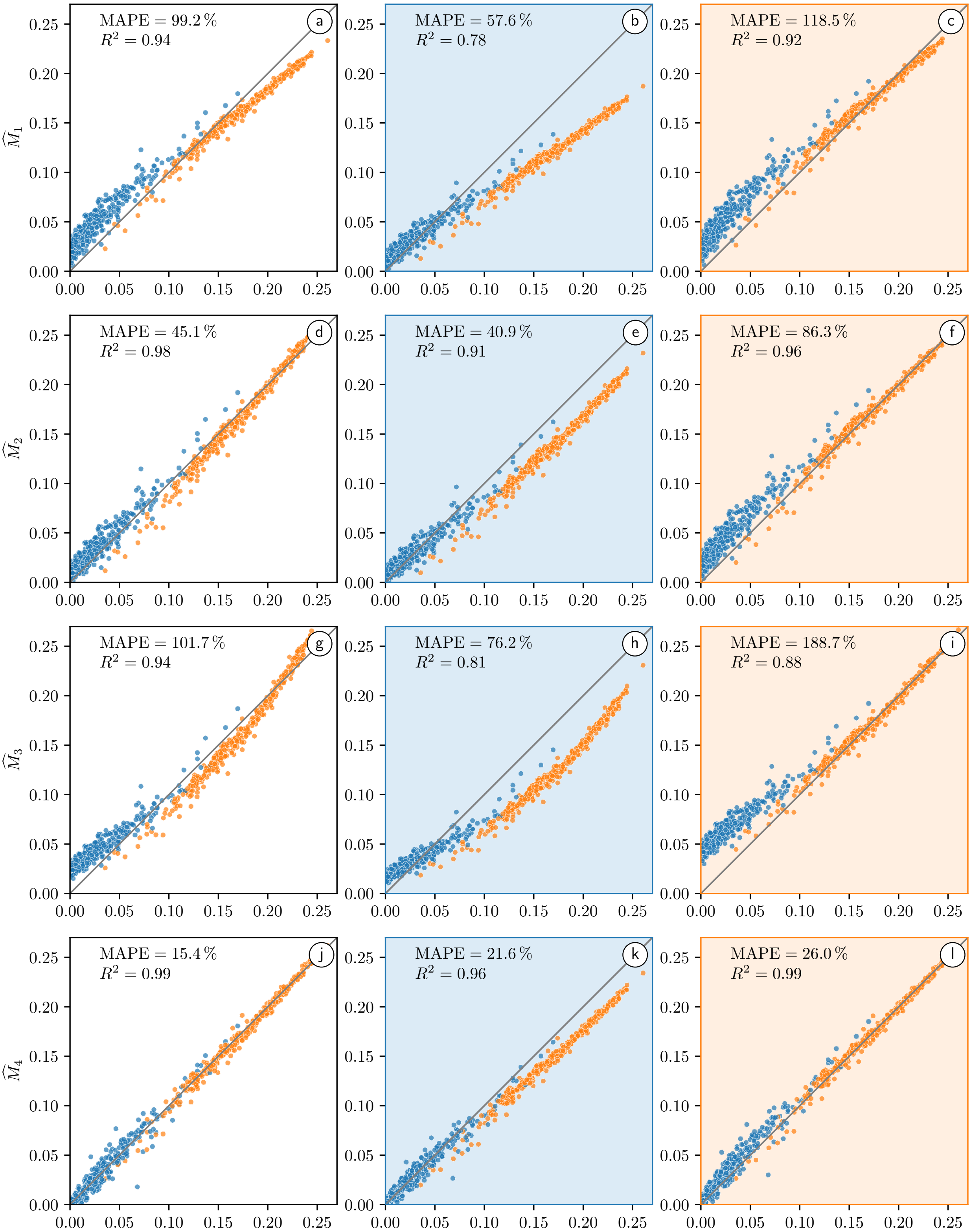


Figure 2: M-factor obtained by numerical simulations versus the predicted M-factor $\widehat{M}_i, i = 1, \ldots, 4$, obtained for the validation data, where different regression formulas (given by Eqs. (1), (2), (3) and (4); from top to bottom) have been used. The colors of data points represent loam (blue) and sand (orange), respectively. The parameter values of regression formulas have been fitted on the entire training data (left), loam (center) and sand (right), respectively.

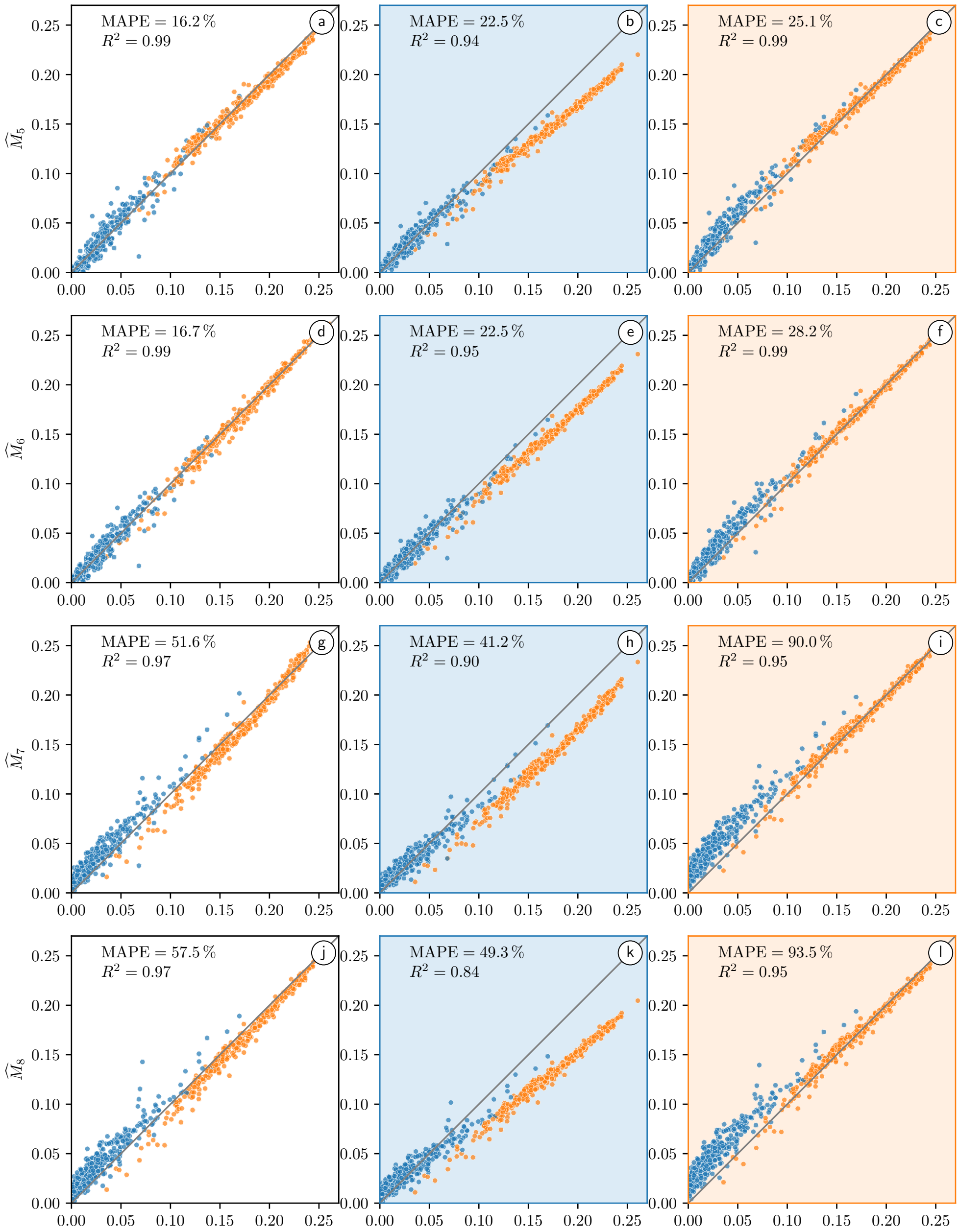


Figure 3: M-factor obtained by numerical simulations versus the predicted M-factor $\widehat{M}_i, i = 5, \ldots, 8$, obtained for the validation data, where different regression formulas (given by Eqs. (5), (6), (7), (8); from top to bottom) have been used. In particular, Eq. (6) is used twice, once for geodesic tortuosity and once for geometric tortuosity. The colors of data points represent loam (blue) and sand (orange), respectively. The parameter values of regression formulas have been fitted on the entire training data (left), loam (center) and sand (right), respectively.

$\widehat{M}_1 = \varepsilon^{c_1}$

| Fitting on | $R^2$: All | $R^2$: Loam | $R^2$: Sand | MAPE [%]: All | MAPE [%]: Loam | MAPE [%]: Sand | $c_1$ |
|---|---|---|---|---|---|---|---|
| All | 0.94 | 0.50 | 0.90 | 99.2 | 192.8 | 6.2 | 1.87 |
| Loam | 0.78 | 0.89 | −0.40 | 57.6 | 86.3 | 29.1 | 2.16 |
| Sand | 0.92 | 0.20 | 0.97 | 118.5 | 233.3 | 4.3 | 1.80 |

Validation on

$\widehat{M}_2 = c_1\varepsilon^{c_2}$

| Fitting on | $R^2$: All | $R^2$: Loam | $R^2$: Sand | MAPE [%]: All | MAPE [%]: Loam | MAPE [%]: Sand | $(c_1, c_2)$ |
|---|---|---|---|---|---|---|---|
| All | 0.98 | 0.85 | 0.95 | 45.1 | 85.1 | 5.4 | $(1.95, 2.53)$ |
| Loam | 0.91 | 0.92 | 0.46 | 40.9 | 62.5 | 19.5 | $(1.69, 2.56)$ |
| Sand | 0.96 | 0.54 | 0.98 | 86.3 | 170.2 | 2.9 | $(1.28, 2.06)$ |

Validation on

$\widehat{M}_3 = c_1 e^{c_2\varepsilon}$

| Fitting on | $R^2$: All | $R^2$: Loam | $R^2$: Sand | MAPE [%]: All | MAPE [%]: Loam | MAPE [%]: Sand | $(c_1, c_2)$ |
|---|---|---|---|---|---|---|---|
| All | 0.94 | 0.57 | 0.89 | 101.7 | 195.6 | 8.4 | $(0.01, 7.41)$ |
| Loam | 0.81 | 0.80 | −0.17 | 76.2 | 124.3 | 28.3 | $(0.01, 7.73)$ |
| Sand | 0.88 | −0.34 | 0.98 | 188.7 | 375.7 | 2.9 | $(0.02, 5.47)$ |

Validation on

$\widehat{M}_4 = \varepsilon^{c_1}\beta^{c_2}\mu(\tau_g)^{c_3}$

| Fitting on | $R^2$: All | $R^2$: Loam | $R^2$: Sand | MAPE [%]: All | MAPE [%]: Loam | MAPE [%]: Sand | $(c_1, c_2, c_3)$ |
|---|---|---|---|---|---|---|---|
| All | 0.99 | 0.92 | 0.98 | 19.5 | 36.0 | 3.1 | $(0.85, -0.35, -11.58)$ |
| Loam | 0.93 | 0.95 | 0.59 | 25.1 | 35.0 | 15.2 | $(1.29, -0.01, -6.84)$ |
| Sand | 0.98 | 0.84 | 0.99 | 38.5 | 74.5 | 2.8 | $(1.27, -0.31, -7.06)$ |

Validation on

$\widehat{M}_5 = \varepsilon^{c_1+c_2\beta}\mu(\tau_g)^{c_3}$

| Fitting on | $R^2$: All | $R^2$: Loam | $R^2$: Sand | MAPE [%]: All | MAPE [%]: Loam | MAPE [%]: Sand | $(c_1, c_2, c_3)$ |
|---|---|---|---|---|---|---|---|
| All | 0.99 | 0.93 | 0.98 | 16.2 | 29.1 | 3.4 | $(1.22, -0.62, -9.23)$ |
| Loam | 0.94 | 0.96 | 0.61 | 22.5 | 30.2 | 14.8 | $(1.67, -0.44, -5.34)$ |
| Sand | 0.99 | 0.89 | 0.98 | 25.1 | 47.0 | 3.4 | $(1.45, -0.51, -6.19)$ |

Validation on

$\widehat{M}_6 = c_1\mu(\tau)^{c_2}\sigma(\tau)^{c_3}\varepsilon^{c_4}$

| Fitting on | $R^2$: All | $R^2$: Loam | $R^2$: Sand | MAPE [%]: All | MAPE [%]: Loam | MAPE [%]: Sand | $(c_1, c_2, c_3, c_4)$ |
|---|---|---|---|---|---|---|---|
| All | 0.99 | 0.95 | 0.98 | 16.7 | 30.8 | 2.7 | $(1.07, -8.15, -0.04, 1.22)$ |
| Loam | 0.95 | 0.96 | 0.72 | 22.5 | 31.7 | 13.2 | $(1.37, -6.06, 0.02, 1.58)$ |
| Sand | 0.99 | 0.89 | 0.99 | 28.2 | 54.4 | 2.3 | $(1.50, -6.00, 0.03, 1.48)$ |

Validation on

$\widehat{M}_7 = \varepsilon^{c_1}S^{c_2}r_{\mathsf{max}}^{c_3}$

| Fitting on | $R^2$: All | $R^2$: Loam | $R^2$: Sand | MAPE [%]: All | MAPE [%]: Loam | MAPE [%]: Sand | $(c_1, c_2, c_3)$ |
|---|---|---|---|---|---|---|---|
| All | 0.97 | 0.79 | 0.95 | 51.6 | 98.0 | 5.5 | $(3.32, -1.06, -0.88)$ |
| Loam | 0.90 | 0.92 | 0.39 | 41.2 | 61.9 | 20.6 | $(3.13, -0.70, -0.55)$ |
| Sand | 0.95 | 0.50 | 0.99 | 90.0 | 177.7 | 2.8 | $(2.38, -0.42, -0.35)$ |

Validation on

$\widehat{M}_8 = \varepsilon^{c_1}(\mu(C)/\mu(H))^{c_2}$

| Fitting on | $R^2$: All | $R^2$: Loam | $R^2$: Sand | MAPE [%]: All | MAPE [%]: Loam | MAPE [%]: Sand | $(c_1, c_2)$ |
|---|---|---|---|---|---|---|---|
| All | 0.98 | 0.88 | 0.95 | 42.0 | 78.5 | 5.7 | $(2.51, 0.43)$ |
| Loam | 0.96 | 0.92 | 0.80 | 33.6 | 55.0 | 12.3 | $(2.67, 0.47)$ |
| Sand | 0.96 | 0.58 | 0.99 | 83.0 | 163.8 | 2.7 | $(2.06, 0.16)$ |

Validation on

Figure 4: Validation scores $R^2$ and MAPE obtained for the regression formulas considered in the present paper including the values for the corresponding regression parameters, where different data sets have been used for training/fitting and validation.

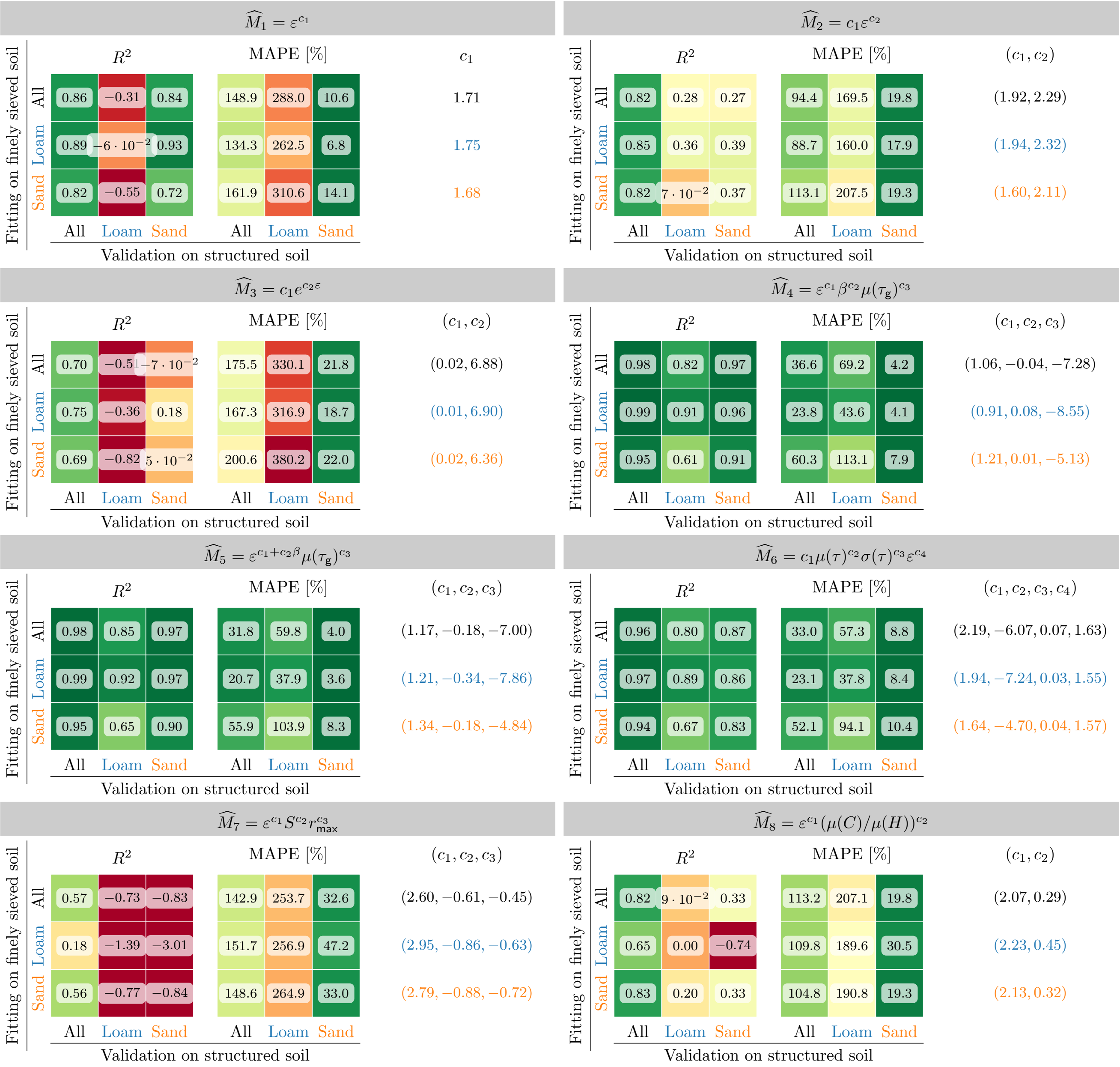


Figure 5: Validation scores $R^2$ and MAPE obtained for the regression formulas considered in the present paper including the values for the corresponding regression parameters. The data set of finely sieved soil described in [1] is used for training/fitting, whereas validation is carried out on image data for structured soil.

| | Formula | $R^2$ | MAPE[%] |
|---|---|---|---|
| $\widehat{M}_1 =$ | $\varepsilon^{1.87}$ | 0.94 | 99.22 |
| $\widehat{M}_2 =$ | $1.95\varepsilon^{2.53}$ | 0.98 | 45.14 |
| $\widehat{M}_3 =$ | $0.01e^{7.41\varepsilon}$ | 0.94 | 101.71 |
| $\widehat{M}_4 =$ | $\varepsilon^{1.26}\beta^{0.11}\mu(\tau_{\mathsf{g}})^{-8.27}$ | 0.99 | 15.41 |
| $\widehat{M}_5 =$ | $\varepsilon^{1.22-0.02\beta}\mu(\tau_{\mathsf{g}})^{-9.23}$ | 0.99 | 16.21 |
| $\widehat{M}_6 =$ | $1.07\mu(\tau)^{-8.15}\sigma(\tau)^{-0.04}\varepsilon^{1.22}$ | 0.99 | 16.72 |
| $\widehat{M}_7 =$ | $\varepsilon^{3.32}S^{-1.06}r_{\mathsf{max}}^{-0.88}$ | 0.97 | 51.63 |
| $\widehat{M}_8 =$ | $\varepsilon^{2.39}(\mu(C)/\mu(H))^{-0.31}$ | 0.97 | 57.52 |

Table 1: Parameter values of regression functions fitted on the entire training data; validation scores $R^2$ and MAPE obtained for the validation data considered in the present paper.

## References

[1] B. Prifling, M. Weber, N. Ray, A. Prechtel, M. Phalempin, S. Schlüter, D. Vetterlein, and V. Schmidt, "Quantifying the impact of 3D pore space morphology on soil gas diffusion in loam and sand" *Transport in Porous Media*, vol. 149, 501–527, 2023.
